# Locality-sensitive bucketing functions for the edit distance

**DOI:** 10.1186/s13015-023-00234-2

**Published:** 2023-07-24

**Authors:** Ke Chen, Mingfu Shao

**Affiliations:** 1 Department of Computer Science and Engineering, The Pennsylvania State University, State College, United States; 2 Huck Institutes of the Life Sciences, The Pennsylvania State University, State College, United States

**Keywords:** Locality-sensitive hashing, Locality-sensitive bucketing, Long reads, Embedding

## Abstract

**Background:**

Many bioinformatics applications involve bucketing a set of sequences where each sequence is allowed to be assigned into multiple buckets. To achieve both high sensitivity and precision, bucketing methods are desired to assign similar sequences into the same bucket while assigning dissimilar sequences into distinct buckets. Existing *k*-mer-based bucketing methods have been efficient in processing sequencing data with low error rates, but encounter much reduced sensitivity on data with high error rates. Locality-sensitive hashing (LSH) schemes are able to mitigate this issue through tolerating the edits in similar sequences, but state-of-the-art methods still have large gaps.

**Results:**

In this paper, we generalize the LSH function by allowing it to hash one sequence into multiple buckets. Formally, a bucketing function, which maps a sequence (of fixed length) into a subset of buckets, is defined to be $(d_1, d_2)$-sensitive if any two sequences within an edit distance of $d_1$ are mapped into at least one shared bucket, and any two sequences with distance at least $d_2$ are mapped into disjoint subsets of buckets. We construct locality-sensitive bucketing (LSB) functions with a variety of values of $(d_1,d_2)$ and analyze their efficiency with respect to the total number of buckets needed as well as the number of buckets that a specific sequence is mapped to. We also prove lower bounds of these two parameters in different settings and show that some of our constructed LSB functions are optimal.

**Conclusion:**

These results lay the theoretical foundations for their practical use in analyzing sequences with high error rates while also providing insights for the hardness of designing ungapped LSH functions.

## Background

Comparing a set of given sequences is a common task involved in many bioinformatics applications, such as homology detection [1], overlap detection and the construction of overlap graphs [2–4], phylogenetic tree reconstruction, and isoform detection from circular consensus sequence (CCS) reads [5], to name a few. The naive all-vs-all comparison gives the most comprehensive information but does not scale well. An efficient and widely-used approach that avoids unnecessary comparisons is *bucketing*: a linear scan is employed to assign each sequence into one or multiple buckets, followed by pairwise comparisons within each bucket. The procedure of assigning sequences into buckets, which we refer to as a *bucketing function*, is desired to be both “sensitive”, i.e., two similar sequences ideally appear in at least one shared bucket so that they can be compared, and “specific”, i.e., two dissimilar sequences ideally appear in disjoint buckets so that they can be exempt from comparison. The criteria of similar/dissimilar sequences are application-dependent; in this work we study bucketing functions for the edit distance (Levenshtein distance).

A simple yet popular bucketing function is to put a sequence into buckets labeled with its own *k*-mers. The popular seed-and-extend strategy [6, 7] implicitly uses this approach. Various sketching methods such as minimizer [8–11] and universal hitting set [12, 13] reduce the number of buckets a sequence is assigned to by only considering a subset of representative *k*-mers. These bucketing methods based on exact *k*-mer matching enjoyed tremendous success in analyzing next-generation sequencing (NGS) data, but are challenged by the third-generation long-reads sequencing data represented by PacBio [14] and Oxford Nanopore [15] technologies; due to the high error rates, sequences that should be assigned to the same buckets hardly share any identical *k*-mers (for a reasonably large *k* such as $k = 21$ with 15% error rate), and therefore results in poor sensitivity.

To address this issue, it is required to be able to recognize similar but not necessarily identical sequences. A general solution is locality-sensitive hashing (LSH) [16, 17] where with high probability, similar sequences are sent into the same bucket (i.e., there is a hash collision), and with high probability dissimilar sequences are sent into different buckets. However, designing locality-sensitive hashing functions for the edit distance is hard; the state-of-the-art method Order Min Hash (OMH) is proved to be a gapped LSH but admits a large gap [16]. Another related approach is embedding the metric space induced by the edit distance into more well-studied normed spaces [4, 18, 19]. However, such an embedding is also hard; for example, it is known that the embedding into $L_1$ cannot be distortion-free [20]. In addition, there are seeding/sketching methods such as spaced *k*-mer [21, 22], indel seeds [23], and the more recent strobemer [24] that allow gaps in the extracted seeds to accommodate some edits, but an edit that happens within the chosen seed can still cause mismatches.

It is worth noting that locality-sensitive hashing functions, when interpreted as bucketing functions, assign a sequence into exactly one bucket: buckets are labeled with hash values, and a sequence is put into the single bucket where it is hashed to. In this work, we propose the concept of *locality-sensitive bucketing* (LSB) functions as a generalization of LSH functions by allowing it to assign a sequence into multiple buckets. Formally, a bucketing function, which maps a sequence (of fixed length) into one or more buckets, is defined to be $(d_1, d_2)$-sensitive if any two sequences within an edit distance of $d_1$ are mapped into at least one shared bucket, and any two sequences with an edit distance at least $d_2$ are mapped into disjoint subsets of buckets. While a stochastic definition by introducing a distribution on a family of bucketing functions can be made in a similar way as the definition of LSH functions, here we focus on this basic, deterministic definition. We design several LSB functions for a variety of values of $(d_1,d_2)$ including both ungapped ($d_2 = d_1 + 1$) and gapped ($d_2 > d_1 + 1$) ones. This demonstrates that allowing one sequence to appear in multiple buckets makes the locality-sensitive properties easier to satisfy. Moreover, our lower bound proof shows that any (1, 2)-sensitive bucketing function must put each sequence (of length *n*) into at least *n* buckets (see Lemma 2), suggesting that certain ungapped locality-sensitive hashing functions, where each sequence is sent to a single bucket, may not exist.

In the following sections, we first introduce the precise definition of LSB functions and propose criteria to measure them. Two different approaches of designing LSB functions are then presented with results summarized in Table 1. We also show experimental studies of the performance of gapped LSB functions.

**Table 1:** Results on $(d_1, d_2)$-sensitive bucketing functions of length-*n* sequence

$(d_1, d_2)$-sensitive	*B*	|*B*|	$|f(\varvec{s})|$	Ref.
(1, 2)	$\{1,\ldots , |B|\}$	$n|\Sigma |^{n-1}$⁎⁎	*n*⁎⁎	Theorem 1
(1, 3)	$\mathcal {S}_n$	$|\Sigma |^n$	$|N_n^1(\varvec{s})|=(|\Sigma |-1)n+1$	Lemma 6
(1, 3)	$B_n^i$	$|\Sigma |^{n-1}$⁎	$${\left\{ \begin{array}{ll}1&{}\text {if } \varvec{s}\in B\\ n&{}\text {if } \varvec{s}\not \in B\end{array}\right. }$$⁎	Lemma 9–11
(3, 5)	$B_n^i$	$|\Sigma |^{n-1}$	$\le |N_n^2(\varvec{s})|$	Theorem 2
$(r, 2r+1)$, $r>1$	$B_n^i$	$|\Sigma |^{n-1}$	$\le |N_n^{r}(\varvec{s})|$	Lemma 8, 10
$(2r-1, 2r+1)$, $r\ge 3$ odd	$\mathcal {S}_n$	$|\Sigma |^n$	$|N_n^r(\varvec{s})|$	Lemma 6
$(2r, 2r+1)$, $r\ge 2$ even	$\mathcal {S}_n$	$|\Sigma |^n$	$|N_n^r(\varvec{s})|$	Lemma 6

Entries with $\le$ show the best known upper bounds. Entries marked with a single star cannot be reduced under the specific bucketing method. Entries marked with double stars cannot be reduced in general. In column *B*, we use $B_n^i$ to refer to a constructed (1, 1)-guaranteed subset

## Basics of locality-sensitive bucketing (LSB) functions

Given an alphabet $\Sigma$ with $|\Sigma |>1$ and a natural number *n*, let $\mathcal {S}_n=\left( \Sigma ^n, \text {edit}\right)$ be the metric space of all length-*n* sequences equipped with the Levenshtein (edit) distance. Given a set *B* of buckets, a bucketing function *f* maps $\mathcal {S}_n$ to $\mathcal {P}(B)$, the power set of *B*. This can be viewed as assigning a sequence $\varvec{s}$ of length *n* to a subset of buckets $f(\varvec{s})\subset B$. Let $d_1 < d_2$ be two non-negative integers, we say a bucketing function *f* is $\left( d_1, d_2\right)$-*sensitive* if for all $\varvec{s}, \varvec{t}\in \mathcal {S}_n$,

1 $$\begin{aligned} \text {edit}\left( \varvec{s}, \varvec{t}\right) \le d_1 \implies f(\varvec{s})\cap f(\varvec{t})\ne \emptyset , \end{aligned}$$

2 $$\begin{aligned} \text {edit}\left( \varvec{s}, \varvec{t}\right) \ge d_2 \implies f(\varvec{s})\cap f(\varvec{t})=\emptyset . \end{aligned}$$

We refer to the above two conditions as LSB-properties (1) and (2) respectively. Intuitively, the LSB-properties state that, if two length-*n* sequences are within an edit distance of $d_1$, then the bucketing function *f* guarantees assigning them to at least one common bucket, and if two length-*n* sequences have an edit distance at least $d_2$, then the bucketing function *f* guarantees not assigning them to any shared bucket. In other words, $(d_1,d_2)$-sensitive bucketing functions perfectly distinguish length-*n* sequences within distance $d_1$ from those with distances at least $d_2$. It is easy to show that if $f:\mathcal {S}_n\rightarrow \mathcal {P}(B)$ is a $(d_1, d_2)$-sensitive bucketing function, then $f(\varvec{s})\ne \emptyset$ for all $\varvec{s}\in \mathcal {S}_n$. In fact, since $\text {edit}\left( \varvec{s}, \varvec{s}\right) =0\le d_1$, the LSB-property (1) implies that $f(\varvec{s})=f(\varvec{s})\cap f(\varvec{s})\ne \emptyset$. If $d_1=d_2-1$ then we say the bucketing function is ungapped; otherwise it is called gapped.

We note that the above definition of LSB functions generalizes the (deterministic) LSH functions: if we require that $|f(\varvec{s})| = 1$ for every sequence $\varvec{s}\in \mathcal {S}_n$, i.e., *f* maps a sequence to a single bucket, then $f(\varvec{s})\cap f(\varvec{t})\ne \emptyset$ implies $f(\varvec{s}) = f(\varvec{t})$ and $f(\varvec{s})\cap f(\varvec{t}) = \emptyset$ implies $f(\varvec{s})\ne f(\varvec{t})$.

Two related parameters can be used to measure an LSB function: |*B*|, the total number of buckets, and $|f(\varvec{s})|$, the number of different buckets that contain a specific sequence $\varvec{s}$. From a practical perspective, it is desirable to keep both parameters small. We therefore aim to design LSB functions that minimize |*B*| and $|f(\varvec{s})|$. Specifically, in the following sections, we will construct $(d_1, d_2)$-sensitive bucketing functions with a variety of values of $(d_1,d_2)$, and analyze their corresponding |*B*| and $|f(\varvec{s})|$; we will also prove lower bounds of |*B*| and $|f(\varvec{s})|$ in different settings and show that some of our constructed LSB functions are optimal, in terms of minimizing these two parameters.

The bounds of |*B*| and $|f(\varvec{s})|$ are closely related to the structure of the metric space $\mathcal {S}_n$. For a sequence $\varvec{s}\in \mathcal {S}_n$, its *d*-neighborhood, denoted by $N_n^d(\varvec{s})$, is the subspace of all sequences of length *n* with edit distance at most *d* from $\varvec{s}$; formally $N_n^d(\varvec{s}) = \{\varvec{t} \in \mathcal {S}_n \mid \text {edit}(\varvec{s}, \varvec{t}) \le d\}$. The following simple fact demonstrates the connection between the bound of $|f(\varvec{s})|$ and the structure of $\mathcal {S}_n$, which will be used later.

### **Lemma 1**

*Let* $\varvec{s}$ *be a sequence of length n. If* $N_n^{d_1}(\varvec{s})$ *contains a subset X with* $|X|=x$ *such that every two sequences in X have an edit distance at least* $d_2$*, then for any* $(d_1,d_2)$*-sensitive bucketing function f we must have *$|f(\varvec{s})| \ge x$.

### Proof

Let *f* be an arbitrary $(d_1,d_2)$-sensitive bucketing function. By the LSB-property (2), the *x* sequences in *X* must be assigned to distinct buckets by *f*. On the other hand, since they are all in $N_n^{d_1}(\varvec{s})$, the LSB-property (1) requires that $f(\varvec{s})$ overlaps with $f(\varvec{t})$ for each sequence $\varvec{t}\in X$. Combined, we have $|f(\varvec{s})| \ge x$. $\square$

## An optimal (1, 2)-sensitive bucketing function

In the most general setting of LSB functions, the labels of buckets in *B* are just symbols that are irrelevant to the construction of the bucketing function. Hence we can let $B=\{1, \ldots , |B|\}$. The remaining of this section studies (1, 2)-sensitive bucketing functions in this general case. We first prove lower bounds of |*B*| and $|f(\varvec{s})|$ in this setting; we then give algorithms to construct an optimal (1, 2)-sensitive bucketing function *f* that matches these bounds.

### Lemma 2

If $f: \mathcal {S}_n \rightarrow \mathcal {P}(B)$ is (1, 2)-sensitive, then for each $\varvec{s}\in \mathcal {S}_n$, $|f(\varvec{s})|\ge n$.

### Proof

According to Lemma 1 with $d_1 = 1$ and $d_2 = 2$, we only need to show that $N_n^1(\varvec{s})$ contains *n* different sequences with pairwise edit distances at least 2. For $i=1, \ldots , n$, let $\varvec{t}^i$ be a sequence obtained from $\varvec{s}$ by a single substitution at position *i*. If $i\ne j$, then $\varvec{t}^i$ differs from $\varvec{t}^j$ at two positions, namely *i* and *j*. Then we must have $\text {edit}\left( \varvec{t}^i, \varvec{t}^j\right) \ge 2$ as $\varvec{t}^i$ cannot be transformed into $\varvec{t}^j$ with a single substitution or a single insertion or deletion. Hence, $\left\{ \varvec{t}^1,\ldots , \varvec{t}^n\right\}$ forms the required set. $\square$

### Lemma 3

If $f: \mathcal {S}_n \rightarrow \mathcal {P}(B)$ is (1, 2)-sensitive, then $|B|\ge n|\Sigma |^{n-1}$.

### Proof

Consider the collection of pairs $H=\left\{ (\varvec{s}, b) \,|\, {\varvec{s}}\in {\mathcal {S}}_n{\text { and }} b\in f({\varvec{s}}) \right\}$. We bound the size of *H* from above and below. For an arbitrary sequence $\varvec{s}$, let $b\in f(\varvec{s})$ be a bucket that contains $\varvec{s}$. According to the LSB-property (2), any other sequence in *b* has edit distance 1 from $\varvec{s}$, i.e., a substitution. Suppose that the bucket *b* contains two sequences $\varvec{u}$ and $\varvec{v}$ that are obtained from $\varvec{s}$ by a single substitution at different positions. Then $\text {edit}\left( \varvec{u}, \varvec{v}\right) =2$ and $f(\varvec{u})\cap f(\varvec{v})\ne \emptyset$, which contradicts the LSB-property (2). Therefore, all the sequences in *b* can only differ from $\varvec{s}$ at some fixed position *i*. There are $|\Sigma |$ such sequences (including $\varvec{s}$ itself). So each bucket $b\in B$ can appear in at most $|\Sigma |$ pairs in *H*. Thus $|H|\le |\Sigma |\cdot |B|$.

On the other hand, according to Lemma 2, each $\varvec{s}\in \mathcal {S}_n$ needs to appear in at least *n* different buckets, and hence at least *n* pairs in *H*. So $|H|\ge n |\mathcal {S}_n|=n|\Sigma |^n$. Together, we have $|\Sigma |\cdot |B|\ge n |\Sigma |^n$, or $|B|\ge n |\Sigma |^{n-1}$. $\square$

We now construct a bucketing function $f:\mathcal {S}_n\rightarrow \mathcal {P}(B)$ that is (1, 2)-sensitive using the algorithm given below. It has exponential running time with respect to *n* but primarily serves as a constructive proof that (1, 2)-sensitive bucketing functions exist. Assign to the alphabet $\Sigma$ an arbitrary order $\sigma :\{1, \ldots , |\Sigma |\}\rightarrow \Sigma$ (for conciseness, we also write $\sigma _i=\sigma (i)$ and assume the inverse function $\sigma ^{-1}(\sigma _i) = i$).
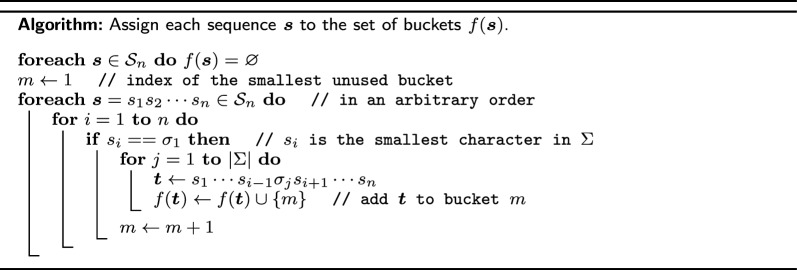


A toy example of the bucketing function *f* with $n=2$ and $\Sigma =\{\sigma _1=\textrm{A},\sigma _2=\textrm{C}, \sigma _3=\textrm{G},\sigma _4=\textrm{T}\}$ constructed using the above algorithm (where the sequences are processed in the lexicographical order induced by $\sigma$) is given below, followed by the contained sequences in the resulting buckets.

**Table:** 

$f(\textrm{AA})=\{1, 2\}$,	$f(\textrm{AC})=\{2, 3\}$,	$f(\textrm{AG})=\{2, 4\}$,	$f(\textrm{AT})=\{2, 5\}$,
$f(\textrm{CA})=\{1, 6\}$,	$f(\textrm{CC})=\{3, 6\}$,	$f(\textrm{CG})=\{4, 6\}$,	$f(\textrm{CT})=\{5, 6\}$,
$f(\textrm{GA})=\{1, 7\}$,	$f(\textrm{GC})=\{3, 7\}$,	$f(\textrm{GG})=\{4, 7\}$,	$f(\textrm{GT})=\{5, 7\}$,
$f(\textrm{TA})=\{1, 8\}$,	$f(\textrm{TC})=\{3, 8\}$,	$f(\textrm{TG})=\{4, 8\}$,	$f(\textrm{TT})=\{5, 8\}$.

**Table:** 

bucket #	sequences	bucket #	sequences
1	AA, CA, GA, TA	2	AA, AC, AG, AT
3	AC, CC, GC, TC	4	AG, CG, GG, TG
5	AT, CT, GT, TT	6	CA, CC, CG, CT
7	GA, GC, GG, GT	8	TA, TC, TG, TT

### Lemma 4

The constructed bucketing function $f:\mathcal {S}_n\rightarrow \mathcal {P}(B)$ satisfies: (i) each bucket contains $|\Sigma |$ sequences, (ii) $|f(\varvec{s})|=n$ for each $\varvec{s}\in \mathcal {S}_n$, and (iii) $|B|=n|\Sigma |^{n-1}$.

### Proof

Claim (i) follows directly from the construction (the most inner for-loop). In the algorithm, each sequence $\varvec{s} \in \mathcal {S}_n$ is added to *n* different buckets, one for each position. Specifically, let $\varvec{s} = s_1s_2\cdots s_n$, then $\varvec{s}$ is added to a new bucket when we process the sequence $\varvec{s}^i = s_1 s_2 \cdots s_{i-1} \sigma _1 s_{i+1} \cdots s_n$, $1 \le i \le n$. Hence, $|f(\varvec{s})|=n$. To calculate |*B*|, observe that a new bucket is used whenever we encounter the smallest character $\sigma _1$ in some sequence $\varvec{s}$. So |*B*| is the same as the number of occurrences of $\sigma _1$ among all sequences in $\mathcal {S}_n$. The total number of characters in $\mathcal {S}_n$ is $n|\Sigma |^n$. By symmetry, $\sigma _1$ appears $n|\Sigma |^{n-1}$ times. $\square$

### Lemma 5

The constructed bucketing function *f* is (1, 2)-sensitive.

### Proof

We show that for $\varvec{s}, \varvec{t}\in \mathcal {S}_n$, $\text {edit}\left( \varvec{s}, \varvec{t}\right) \le 1$ if and only if $f(\varvec{s})\cap f(\varvec{t})\ne \emptyset$. For the forward direction, $\text {edit}\left( \varvec{s}, \varvec{t}\right) \le 1$ implies that $\varvec{s}$ and $\varvec{t}$ can differ by at most one substitution at some position *i*. Let $\varvec{r}$ be the sequence that is identical to $\varvec{s}$ except at the *i*-th position where it is substituted by $\sigma _1$ (it is possible that $\varvec{r} = \varvec{s}$). According to the algorithm, when processing $\varvec{r}$, both $\varvec{s}$ and $\varvec{t}$ are added to a same bucket *m*. Therefore, $m\in f(\varvec{s})\cap f(\varvec{t})$.

For the backward direction, let *m* be an integer from $f(\varvec{s})\cap f(\varvec{t})$. By construction, all the $|\Sigma |$ sequences in the bucket *m* differ by a single substitution. Hence, $\text {edit}\left( \varvec{s}, \varvec{t}\right) \le 1$. $\square$

Combining Lemmas 2–5, we have shown that the above (1, 2)-sensitive bucketing function is optimal in the sense of minimizing |*B*| and $|f(\varvec{s})|$. This is summarized below.

### Theorem 1

Let $B=\{1, \ldots , n |\Sigma |^{n-1}\}$, there is a (1, 2)-sensitive bucketing function $f:\mathcal {S}_n \rightarrow \mathcal {P}(B)$ with $|f(\varvec{s})| = n$ for each $\varvec{s} \in \mathcal {S}_n$. No (1, 2)-sensitive bucketing function exists if |*B*| is smaller or $|f(\varvec{s})|< n$ for some sequence $\varvec{s}\in \mathcal {S}_n$.

### An efficient construction algorithm

In practice, instead of considering the entire $\mathcal {S}_n$, one is often interested in some specific subset *X*. For example, *X* can be the set of all length-*n* strings that appear in a genome. Given an LSB function *f* on $\mathcal {S}_n$, let $f|_X$ be its restriction to *X*. Then $f|_X$ satisfies the LSB-properties (1) and (2) for all $\varvec{s}, \varvec{t}\in X$. In the case that *X* is much smaller in size comparing to $\mathcal {S}_n$, it is desirable to compute $f|_X$ directly.

The above algorithm constructs an optimal (1, 2)-sensitive bucketing function by assigning *n* buckets to each $\varvec{s}\in \mathcal {S}_n$ with a global counter. It runs in $O\left( n|\Sigma |^n\right)$ time. We now show that the *n* buckets assigned to a sequence $\varvec{s}$ can be computed directly in *O*(*n*) time, implying a *O*(*n*|*X*|)-time algorithm that computes a (1, 2)-sensitive bucketing function for an arbitrary subset $X\subset \mathcal {S}_n$.

Recall that in the above algorithm, a new integer bucket is used whenever we encounter the smallest character $\sigma _{1} \in \Sigma$ in a sequence $\varvec{s}$, then all $|\Sigma |$ sequences with a single mutation at this position, including $\varvec{s}$ itself, are added to this bucket. If the sequences are processed in the lexicographical order induced by $\sigma$, this integer is essentially counting the number of occurrences of $\sigma _{1}$ that come before (in this lexicographical order) the current $\sigma _{1}$. For instance, in the previous example, the character A in AT triggers a new bucket 5 because there are four A’s come before it in the lexicographical order; AT is also in bucket 2 because it can be obtained by a single mutation of AA where the underlined A is the second in the lexicographical order. In general, the sequence $\varvec{s}=s_1s_2\cdots s_n\in \mathcal {S}_n$ is assigned to *n* buckets triggered by the underlined $\sigma _1$’s in the *n* (not necessarily distinct) sequences $\varvec{s}^1 = \underline{\sigma _1}s_2\cdots s_n$, $\varvec{s}^2 = s_1\underline{\sigma _1}s_3\cdots s_n$, $\ldots$, $\varvec{s}^n = s_1\cdots s_{n-1}\underline{\sigma _1}$, respectively.

For $\varvec{t} \in \mathcal {S}_n$, let $S_{\sigma }(\varvec{t})$ be the set of sequences in $\mathcal {S}_n$ that come before $\varvec{t}$ in the lexicographical order induced by $\sigma$, namely, $S_{\sigma }(\varvec{t})$ contains $\sigma _1^n, \sigma _1^{n-1}\sigma _2, \ldots$ up to the length-*n* sequence immediately before $\varvec{t}$. Define $\text {count}(\varvec{t})$ to be the total number of $\sigma _{1}$’s among all sequences in $S_{\sigma }(\varvec{t})$. Let $\#_i^1(\varvec{t})$ be the number of $\sigma _1$’s in the length-*i* prefix of $\varvec{t}$. Then $\varvec{s}$ is added to the buckets $\left\{ \text {count}\left( \varvec{s}^i\right) + \#_{i-1}^1\left( \varvec{s}\right) + 1 \,|\, i=1, \ldots , n\right\}$.

We first consider the computation of $\text {count}\left( \varvec{t}\right) =\text {count}\left( t_1t_2\cdots t_n\right)$. If $t_1=\sigma _1$, then all sequences in $S_{\sigma }(\varvec{t})$ begin with $\sigma _1$, there are $|S_{\sigma }(\varvec{t})|=|S_{\sigma }(t_2\cdots t_n)|$ of them; removing the first character of all the sequences in $S_{\sigma }(\varvec{t})$ produces the set $S_{\sigma }(t_2\cdots t_n)$. So $\text {count}(t_1t_2\cdots t_n) = |S_{\sigma }(\varvec{t})| + \text {count}(t_2\cdots t_n)$. If $t_1\ne \sigma _1$, consider the sequence $\varvec{m} = {\hat{t}}_1 \sigma _{|\Sigma |}^{n-1}$ where ${\hat{t}}_1$ is the character precedes $t_1$ according to $\sigma$. We compute $\text {count}(\varvec{t})$ by partition $S_{\sigma }(\varvec{t})$ into three sets: the set of sequences come before $\varvec{m}$, the set of sequences come after $\varvec{m}$ (but before $\varvec{t}$), and the singleton set $\{\varvec{m}\}$. For the first set, the number of $\sigma _1$ is $\text {count}(\varvec{m})$ by definition. For the second set, all the sequences begin with $t_1\ne \sigma _1$ (note that the sequence immediately after $\varvec{m}$ is $t_1\sigma _1^{n-1}$), so removing the first character does not affect the number of $\sigma _1$’s; observe that this produces the set $S_{\sigma }(t_2\cdots t_n)$. For the third set, the only possible occurrence of $\sigma _1$ is ${\hat{t}}_1$. In summary, $\text {count}(\varvec{t})$ can be computed by the following recursive formula:

$$\begin{aligned} \text {count}\left( t_1t_2\cdots t_n\right) = \text {count}\left( t_2\cdots t_n\right) + {\left\{ \begin{array}{ll} |S_{\sigma }\left( t_2\cdots t_n\right) | &{} \text { if } t_1 = \sigma _{1},\\ \text {count}\left( \sigma _{1} \sigma _{|\Sigma |}^{n-1}\right) + 1 &{} \text { if } t_1 = \sigma _{2},\\ \text {count}\left( {\hat{t}}_1 \sigma _{|\Sigma |}^{n-1}\right) &{} \text { otherwise}, \end{array}\right. } \end{aligned}$$

with base case $\text {count}(\varepsilon ) = 0$ and $S_{\sigma }(\varepsilon )=\emptyset$ where $\varepsilon$ denotes the empty string.

In the first case, the number of length-$(n-1)$ sequences before $t_{2}\cdots t_n$ in the lexicographical order has the closed-form expression (corresponds to the base-$|\Sigma |$ numeral encoding of the sequence $t_{2}\cdots t_n$ with respect to $\sigma$):

3 $$\begin{aligned} |S_{\sigma }(t_{2}\cdots t_n)| = \sum _{j=2}^n \left( \sigma ^{-1}\left( t_j\right) - 1\right) |\Sigma |^{n-j}. \end{aligned}$$

Expanding the second case by the recursion, we have

$$\begin{aligned} \text {count}\left( \sigma _1\sigma _{|\Sigma |}^{n-1}\right) = \text {count}\left( \sigma _{|\Sigma |}^{n-1}\right) + \left| S_{\sigma }\left( \sigma _{|\Sigma |}^{n-1}\right) \right| =(n-1)|\Sigma |^{n-2} + |\Sigma |^{n-1} - 1, \end{aligned}$$

where in the second equation, the first term is by symmetry of all characters from $\left\{ \sigma _1^{n-1}, \ldots , \sigma _{|\Sigma |}^{n-1}\right\} =\mathcal {S}_{n-1}$ (technically, $\text {count}\left( \sigma _{|\Sigma |}^{n-1}\right)$ excludes $\sigma _1$’s from $\sigma _{|\Sigma |}^{n-1}$, but there is none); and the second term is simply $\left| \mathcal {S}_{n-1}\setminus \left\{ \sigma _{|\Sigma |}^{n-1}\right\} \right|$. Expanding the third case by the recursion until the first character becomes $\sigma _1$, we have

$$\begin{aligned} \text {count}\left( {\hat{t}}_1\sigma _{|\Sigma |}^{n-1}\right)&= \text {count}\left( \sigma _{|\Sigma |}^{n-1}\right) + \text {count}\left( \hat{{\hat{t}}}_1\sigma _{|\Sigma |}^{n-1}\right) = \ldots \\&= \left( \sigma ^{-1}\left( {\hat{t}}_1\right) - 1\right) \cdot \text {count}\left( \sigma _{|\Sigma |}^{n-1}\right) + \text {count}\left( \sigma _1\sigma _{|\Sigma |}^{n-1}\right) + 1\\&= \sigma ^{-1}\left( {\hat{t}}_1\right) (n-1)|\Sigma |^{n-2} + |\Sigma |^{n-1}. \end{aligned}$$

For conciseness, define for $i=1, 2,\ldots , n$:

$$\begin{aligned} \mu _i(\varvec{t}) = \mu (t_i\cdots t_n)= {\left\{ \begin{array}{ll} |S_{\sigma }\left( t_{i+1}\cdots t_n\right) | &{} \text { if } t_i = \sigma _{1},\\ \sigma ^{-1}\left( t_i\right) (n-i)|\Sigma |^{n-i-1} + |\Sigma |^{n-i} &{} \text { if } t_i \ne \sigma _{1}. \end{array}\right. } \end{aligned}$$

Then the recursion can be simplified to

$$\begin{aligned} \text {count}\left( \varvec{t}\right) =\text {count}\left( t_1t_2\cdots t_n\right) = \text {count}\left( t_2\cdots t_n\right) + \mu _1(\varvec{t}) =\ldots =\sum _{i=1}^{n}\mu _i(\varvec{t}). \end{aligned}$$

By equation (3), the $\mu _i(\varvec{t})$’s can be computed iteratively from *n* to 1 yielding a linear time algorithm for computing $\text {count}(t_1\cdots t_n)$. (Here we assume that all arithmitic operations involved take constant time.)

For the *n* buckets $\left\{ \text {count}\left( \varvec{s}^i\right) + \#_{i-1}^1\left( \varvec{s}\right) + 1 \,|\, i=1, \ldots , n\right\}$, computing each $\text {count}\left( \varvec{s}^i\right)$ separatedly takes $O\left(n^2\right)$ time in total. We aim to reduce the running time by exploring the similarity between $\text {count}\left( \varvec{s}\right)$ and $\text {count}\left( \varvec{s}^i\right)$. For $j<i$, consider $\mu _j(\varvec{s}) = \mu \left( s_j\cdots s_{i-1}s_is_{i+1}\cdots s_n\right)$ and $\mu _j\left( \varvec{s}^i\right) = \mu \left( s_j\cdots s_{i-1}\sigma _1s_{i+1}\cdots s_n\right)$, if $s_j=\sigma _1$, according to equation (3), their values differ by $(\sigma ^{-1}(s_i)-1)|\Sigma |^{n-i}$; and if $s_j\ne \sigma _1$, they are the same by definition. Recall that the number of occurrences of $\sigma _1$ among the first $i-1$ characters in $\varvec{s}$ is $\#_{i-1}^1(\varvec{s})$, hence the values of $\sum _{j=1}^{i-1}\mu _j(\varvec{s})$ and $\sum _{j=1}^{i-1}\mu _j\left( \varvec{s}^i\right)$ differ by $\#_{i-1}^1(\varvec{s})(\sigma ^{-1}(s_i)-1)|\Sigma |^{n-i}$. For $j>i$, $\mu _j(\varvec{s})=\mu _j\left( \varvec{s}^i\right)$ because the two suffixes starting from position *j* are identical. Therefore, we have

$$\begin{aligned} \text {count}\left( \varvec{s}^i\right)&= \sum _{j=1}^{i-1}\mu _j\left( \varvec{s}^i\right) + \mu _i\left( \varvec{s}^i\right) + \sum _{j=i+1}^n\mu _j\left( \varvec{s}^i\right) \\&= \sum _{j=1}^{i-1}\mu _j(\varvec{s}) -\#_{i-1}^1(\varvec{s}) (\sigma ^{-1}(s_i)-1)|\Sigma |^{n-i} + \mu _i\left( \varvec{s}^i\right) + \sum _{j=i+1}^{n}\mu _j(\varvec{s})\\&=\sum _{j=1}^n\mu _j\left( \varvec{s}\right) - \mu _i\left( \varvec{s}\right) -\#_{i-1}^1(\varvec{s}) (\sigma ^{-1}(s_i)-1)|\Sigma |^{n-i} + \mu _i\left( \varvec{s}^i\right) \\&= \text {count}(\varvec{s}) - \mu _i(\varvec{s}) -\#_{i-1}^1(\varvec{s}) (\sigma ^{-1}(s_i)-1)|\Sigma |^{n-i} + |S_{\sigma }(s_{i+1}\cdots s_n)|. \end{aligned}$$

The following pseudocode first calculates and stores in linear time and space the values of $\mu _i(\varvec{s})$, $\#_i^1(\varvec{s})$, and $\nu _i(\varvec{s}) = |S_\sigma (s_{i}\cdots s_n)|$; then each of the *n* buckets is computed in constant time. We also provide an implementation of both the global counter algorithm and this efficient individual bucketing algorithm at [25].
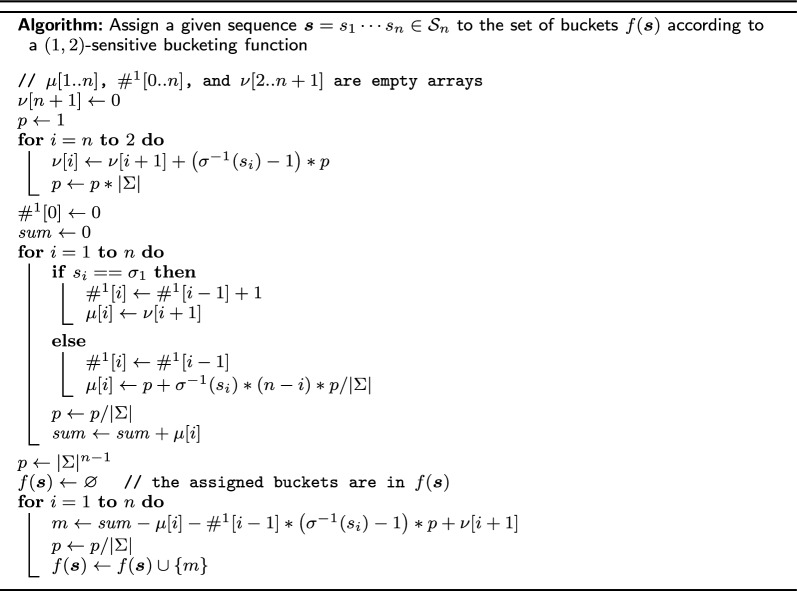


## Mapping to sequences of length *n*

We continue to explore LSB functions with different values of $d_1$ and $d_2$. Here we focus on a special case where $B\subset \mathcal {S}_n$, namely, each bucket in *B* is labeled by a length-*n* sequence. The idea of designing such LSB functions is to map a sequence $\varvec{s}$ to its neighboring sequences that are in *B*. Formally, given a subset $B\subset \mathcal {S}_n$ and an integer $r\ge 1$, we define the bucketing function $f^r_B:\mathcal {S}_n\rightarrow \mathcal {P}(B)$ by

$$\begin{aligned} f^r_B(\varvec{s})= N_n^r(\varvec{s}) \cap B = \left\{ \varvec{v}\in B\,|\, \text {edit}\left( \varvec{s}, \varvec{v}\right) \le r\right\} \text { for each } \varvec{s}\in \mathcal {S}_n. \end{aligned}$$

We now derive the conditions for $f^r_B$ to be an LSB function. For any sequence $\varvec{s}$, all the buckets in $f^r_B(\varvec{s})$ are labeled by its neighboring sequences within radius *r*. Therefore, if two sequences $\varvec{s}$ and $\varvec{t}$ share a bucket labeled by $\varvec{v}$, then $\text {edit}\left( \varvec{s}, \varvec{v}\right) \le r$ and $\text {edit}\left( \varvec{t}, \varvec{v}\right) \le r$. Recall that $\mathcal {S}_n$ is a metric space, in particular, the triangle inequality holds. So $\text {edit}\left( \varvec{s}, \varvec{t}\right) \le \text {edit}\left( \varvec{s}, \varvec{v}\right) +\text {edit}\left( \varvec{t}, \varvec{v}\right) \le 2r$. In other words, if $\varvec{s}$ and $\varvec{t}$ are $2r + 1$ edits apart, then they will be mapped to disjoint buckets. Formally, if $\text {edit}\left( \varvec{s}, \varvec{t}\right) \ge 2r + 1$, then $f^r_B(\varvec{s}) \cap f^r_B(\varvec{t}) = \emptyset$. This implies that $f^r_B$ satisfies the LSB-property (2) with $d_2 = 2r + 1$. We note that this statement holds regardless of the choice of *B*.

Hence, to make $f^r_B$ a $(d_1, 2r+1)$-sensitive bucketing function for some integer $d_1$, we only need to determine a subset *B* so that $f^r_B$ satisfies the LSB-property (1). Specifically, *B* should be picked such that for any two length-*n* sequences $\varvec{s}$ and $\varvec{t}$ within an edit distance of $d_1$, we always have

$$\begin{aligned} f_B^r(\varvec{s}) \cap f_B^r(\varvec{t}) = \left( N_n^r(\varvec{s})\cap B\right) \cap \left( N_n^r(\varvec{t}) \cap B\right) = N_n^r(\varvec{s}) \cap N_n^r(\varvec{t}) \cap B \ne \emptyset . \end{aligned}$$

For the sake of simplicity, we say a set of buckets $B\subset \mathcal {S}_n$ is $(d_1,r)$-*guaranteed* if and only if $N_n^r(\varvec{s}) \cap N_n^r(\varvec{t}) \cap B \ne \emptyset$ for every pair of sequences $\varvec{s}$ and $\varvec{t}$ with $\text {edit}\left( \varvec{s}, \varvec{t}\right) \le d_1$. Equivalently, following the above arguments, *B* is $(d_1,r)$-guaranteed if and only if the corresponding bucketing function $f_B^r$ is $(d_1, 2r+1)$-sensitive. Note that the $(d_1, r)$-guaranteed set is not a new concept, but rather an abbreviation to avoid repeating the long phrase “a set whose corresponding bucketing function is guaranteed to be $(d_1, 2r+1)$-sensitive”. In the following sections, we show several $(d_1, r)$-guaranteed subsets $B\subset \mathcal {S}_n$ for different values of $d_1$.

### (2*r*, *r*)-guaranteed and $(2r - 1, r)$-guaranteed subsets

We first consider an extreme case where $B = \mathcal {S}_n$.

#### Lemma 6

Let $B = \mathcal {S}_n$. Then *B* is (2*r*, *r*)-guaranteed if *r* is even, and *B* is $(2r-1,r)$-guaranteed if *r* is odd.

#### Proof

First consider the case that *r* is even. Let $\varvec{s}$ and $\varvec{t}$ be two length-*n* sequences with $\text {edit}\left( \varvec{s}, \varvec{t}\right) \le 2r$. Then there are 2*r* edits that transforms $\varvec{s}$ to $\varvec{t}$. (If $\text {edit}\left( \varvec{s}, \varvec{t}\right) <2r$, we can add in trivial edits that substitute a character with itself.) Because $\varvec{s}$ and $\varvec{t}$ have the same length, these 2*r* edits must contain the same number of insertions and deletions. Reorder the edits so that each insertion is followed immediately by a deletion (i.e., a pair of indels) and all the indels come before substitutions. Because *r* is even, in this new order, the first *r* edits contain an equal number of insertions and deletions. Namely, applying the first *r* edits on $\varvec{s}$ produces a length-*n* sequence $\varvec{v}$. Clearly, $\text {edit}\left( \varvec{s}, \varvec{v}\right) \le r$ and $\text {edit}\left( \varvec{t}, \varvec{v}\right) \le r$, i.e., $\varvec{v}\in N_n^r(\varvec{s})\cap N_n^r(\varvec{t})= N_n^r(\varvec{s})\cap N_n^r(\varvec{t})\cap B$.

For the case that *r* is odd. Let $\varvec{s}$ and $\varvec{t}$ be two length-*n* sequences with $\text {edit}\left( \varvec{s}, \varvec{t}\right) \le 2r-1$. By the same argument as above, $\varvec{s}$ can be transformed to $\varvec{t}$ by $2r-1$ edits and we can assume that all the indels appear in pairs and they come before all the substitutions. Because *r* is odd, $r-1$ is even. So applying the first $r-1$ edits on $\varvec{s}$ produces a length-*n* sequence $\varvec{v}$ such that $\text {edit}\left( \varvec{s}, \varvec{v}\right) \le r-1<r$ and $\text {edit}\left( \varvec{t}, \varvec{v}\right) \le 2r-1-(r-1)=r$. Therefore, $\varvec{v}\in N_n^r(\varvec{s})\cap N_n^r(\varvec{t})= N_n^r(\varvec{s})\cap N_n^r(\varvec{t})\cap B$. $\square$

By definition, setting $B=\mathcal {S}_n$ makes $f^r_B$ $(2r, 2r+1)$-sensitive if *r* is even and $(2r-1, 2r+1)$-sensitive if *r* is odd. This provides nearly optimal bucketing performance in the sense that there is no gap (when *r* is even) or the gap is just one (when *r* is odd). It is evident from the proof that the gap at 2*r* indeed exists when *r* is odd because if $\varvec{s}$ can only be transformed to $\varvec{t}$ by *r* pairs of indels, then there is no length-*n* sequence $\varvec{v}$ with $\text {edit}\left( \varvec{s}, \varvec{v}\right) =\text {edit}\left( \varvec{t}, \varvec{v}\right) =r$.

### Properties of (*r*, *r*)-guaranteed subsets

In the above section all sequences in $\mathcal {S}_n$ are used as buckets. A natural question is, can we use a proper subset of $\mathcal {S}_n$ to achieve (gapped) LSB functions? This can be viewed as down-sampling $\mathcal {S}_n$ such that if two length-*n* sequences $\varvec{s}$ and $\varvec{t}$ are similar, then a length-*n* sequence is always sampled from their common neighborhood $N_n^r(\varvec{s})\cap N_n^r(\varvec{t})$.

Here we focus on the case that $d_1 = r$, i.e., we aim to construct *B* that is (*r*, *r*)-guaranteed. Recall that this means for any $\varvec{s}, \varvec{t}\in \mathcal {S}_n$ with $\text {edit}\left( \varvec{s}, \varvec{t}\right) \le r$, we have $N_n^r(\varvec{s})\cap N_n^r(\varvec{t})\cap B\ne \emptyset$. In other words, $f^r_B$ is $(r, 2r+1)$-sensitive. To prepare the construction, we first investigate some structural properties of (*r*, *r*)-guaranteed subsets. We propose a conjecture that such sets form a hierarchical structure with decreasing *r*:

#### Conjecture 1

If $B\subset \mathcal {S}_n$ is (*r*, *r*)-guaranteed, then *B* is also $(r+1,r+1)$-guaranteed.

We prove a weaker statement:

#### Lemma 7

If $B\subset \mathcal {S}_n$ is (*r*, *r*)-guaranteed, then *B* is $(r+2,r+2)$-guaranteed.

#### Proof

Let $\varvec{s}$ and $\varvec{t}$ be two length-*n* sequences with $\text {edit}\left( \varvec{s}, \varvec{t}\right) \le r+2$; we want to show that $N_n^{r+2}(\varvec{s}) \cap N_n^{r+2}(\varvec{t}) \cap B \ne \emptyset$. Consider a list of edits that transforms $\varvec{s}$ to $\varvec{t}$: skipping a pair of indels or two substitutions gives a length-*n* sequence $\varvec{m}$ such that $\text {edit}\left( \varvec{s}, \varvec{m}\right) \le r$ and $\text {edit}\left( \varvec{t}, \varvec{m}\right) =2$. Because $\varvec{s}$ and $\varvec{m}$ are within a distance of *r* and *B* is (*r*, *r*)-guaranteed, we have that $N_n^r(\varvec{s}) \cap N_n^r(\varvec{m}) \cap B \ne \emptyset$, i.e., there exists a length-*n* sequence $\varvec{v}\in B$ such that $\text {edit}\left( \varvec{s}, \varvec{v}\right) \le r$ and $\text {edit}\left( \varvec{m}, \varvec{v}\right) \le r$. By triangle inequality, $\text {edit}\left( \varvec{t}, \varvec{v}\right) \le \text {edit}\left( \varvec{t}, \varvec{m}\right) +\text {edit}\left( \varvec{m}, \varvec{v}\right) \le r+2$. Hence, we have $\varvec{v}\in N_n^{r+2}(\varvec{t})$. Clearly, $\varvec{v}\in N_n^{r}(\varvec{s})$ implies that $\varvec{v}\in N_n^{r+2}(\varvec{s})$. Combined, we have $\varvec{v}\in N_n^{r+2}(\varvec{s})\cap N_n^{r+2}(\varvec{t})\cap B$. $\square$

The next lemma shows that (1, 1)-guaranteed subsets have the strongest condition.

#### Lemma 8

If $B\subset S_n$ is (1, 1)-guaranteed, then *B* is (*r*, *r*)-guaranteed for all $r\ge 1$.

#### Proof

According to the previous lemma, we only need to show that *B* is (2, 2)-guaranteed. Given two length-*n* sequences $\varvec{s}$ and $\varvec{t}$ with $\text {edit}\left( \varvec{s}, \varvec{t}\right) =2$, consider a list *Q* of two edits that transforms $\varvec{s}$ to $\varvec{t}$. There are two possibilities:

- If both edits in *Q* are substitutions, let *i* be the position of the first substitution.
- If *Q* consists of one insertion and one deletion, let *i* be the position of the character that is going to be deleted from $\varvec{s}$.

In either case, let $\varvec{m}$ be a length-*n* sequence obtained by replacing the *i*-th character of $\varvec{s}$ with another character in $\Sigma$. Then $\text {edit}\left( \varvec{s}, \varvec{m}\right) =1$. Because *B* is (1, 1)-guaranteed, there is a length-*n* sequence $\varvec{v}\in B$ such that $\text {edit}\left( \varvec{s}, \varvec{v}\right) \le 1$ and $\text {edit}\left( \varvec{m}, \varvec{v}\right) \le 1$. Observe that either $\varvec{s}=\varvec{v}$ or $\varvec{v}$ is obtained from $\varvec{s}$ by one substitution at position *i*. So applying the two edits in *Q* on $\varvec{v}$ also produces $\varvec{t}$, i.e., $\text {edit}\left( \varvec{t}, \varvec{v}\right) \le 2$. Therefore, $\varvec{v}\in N_n^{2}(\varvec{s})\cap N_n^{2}(\varvec{t})\cap B$. $\square$

Now we bound the size of a (1, 1)-guaranteed subset from below.

#### Lemma 9

If *B* is (1,1)-guaranteed, then

$$\begin{aligned} \text {(i) for each } \varvec{s}\in \mathcal {S}_n,\; \left| N_n^1(\varvec{s})\cap B\right| \ge {\left\{ \begin{array}{ll} 1 &{} \text {if }\varvec{s}\in B\\ n &{} \text {if }\varvec{s}\not \in B \end{array}\right. }, \quad \text {(ii) } |B| \ge |\mathcal {S}_n|/|\Sigma | = |\Sigma |^{n-1}. \end{aligned}$$

#### Proof

Let $B\subset \mathcal {S}_n$ be an arbitrary (1, 1)-guaranteed subset. For part (i), because $\varvec{s}\in N_n^1(\varvec{s})$, if $\varvec{s}$ is also in *B*, then $\varvec{s}$ is in their intersection, hence $\left| N_n^1(\varvec{s})\cap B\right| \ge 1$. If $\varvec{s}=s_1s_2\ldots s_n \not \in B$, then it must have at least *n* 1-neighbors $\varvec{v}^i\in B$, one for each position $1\le i\le n$, where $\varvec{v}^i = s_1\ldots s_{i-1}v_is_{i+1}\ldots s_n$, $v_i\ne s_i$. Suppose conversely that this is not the case for a particular *i*. Let $\varvec{t}= s_1\ldots s_{i-1}t_is_{i+1}\ldots s_n$ where $t_i\ne s_i$. We have $\text {edit}\left( {\varvec{s}}, {\varvec{t}}\right) =1$. Also, $N_n^1({\varvec{s}})\cap N_n^1({\varvec{t}}) = \{x\in \Sigma \mid s_1\ldots s_{i-1}xs_{i+1}\ldots s_n\}$, but none of them is in *B* (consider the two cases $x=s_i$ and $x\ne s_i$), i.e., $N_n^1(\varvec{s})\cap N_n^1(\varvec{t}) \cap B = \emptyset$. This contradicts the assumption that *B* is (1, 1)-guaranteed.

For part (ii), consider the set of pairs $H=\left\{ (\varvec{s}, \varvec{v})\,\mid \, \varvec{s}\in \mathcal {S}_n \text { and } \varvec{v}\in N_n^1(\varvec{s})\cap B \right\}$. For all $\varvec{v}\in B$, the number of sequences $\varvec{s}\in \mathcal {S}_n$ with $\text {edit}\left( \varvec{s}, \varvec{v}\right) \le 1$ is $n\left( |\Sigma |-1\right) +1$. So $|H| = \left( n\left( |\Sigma |-1\right) +1\right) |B|$. On the other hand, part (i) implies that $|H|\ge |B|+ n\left( |\Sigma |^n-|B|\right)$. Combined, we have $|B|\ge |\Sigma |^{n-1}$, as claimed. $\square$

Next, we give an algorithm to construct a (1, 1)-guaranteed subset *B* that achieves the size $|B| = |\Sigma |^{n-1}$; furthermore, the corresponding (1, 3)-sensitive bucketing function $f^1_B$ satisfies $\left| f^1_B(\varvec{s})\right| =1$ if $\varvec{s}\in B$ and $\left| f^1_B(\varvec{s})\right| =n$ if $\varvec{s}\not \in B$. This shows that the lower bounds proved above in Lemma 9 are tight and that the constructed (1, 1)-guaranteed subset *B* is optimal in the sense of minimizing both |*B*| and $\left| f^1_B(\varvec{s})\right|$. Notice that this result improves Lemma 6 with $r = 1$ where we showed that $\mathcal {S}_n$ is a (1, 1)-guaranteed subset of size $|\Sigma |^n$. According to Lemma 8, this constructed *B* is also (*r*, *r*)-guaranteed. So the corresponding bucketing function $f_B^r$ is $(r, 2r+1)$-sensitive for all integers $r\ge 1$.

### Construction of optimal (1, 1)-guaranteed subsets

Let $m=|\Sigma |$ and denote the characters in $\Sigma$ by $c_1, c_2, \ldots , c_m$. We describe a recursive procedure to construct a (1, 1)-guaranteed subset of $\mathcal {S}_n$. In fact, we show that $\mathcal {S}_n$ can be partitioned into *m* subsets $B_n^1 \sqcup B_n^2\sqcup \cdots \sqcup B_n^m$ such that each $B_n^i$ is (1, 1)-guaranteed. Here the notation $\sqcup$ denotes disjoint union. The partition of $\mathcal {S}_{n}$ is built from the partition of $\mathcal {S}_{n-1}$. The base case is $\mathcal {S}_1=\{c_1\}\sqcup \cdots \sqcup \{c_m\}$.

Suppose that we already have the partition for $\mathcal {S}_{n-1} = B_{n-1}^1 \sqcup B_{n-1}^2\sqcup \cdots \sqcup B_{n-1}^m$. Let

$$\begin{aligned} B_n^1=\left( c_1\circ B_{n-1}^1\right) \sqcup \left( c_2\circ B_{n-1}^2\right) \sqcup \cdots \sqcup \left( c_m\circ B_{n-1}^{m}\right) , \end{aligned}$$

where $c\circ B$ is the set obtained by prepending the character *c* to each sequence in the set *B*. For $B_n^2$, the construction is similar where the partitions of $\mathcal {S}_{n-1}$ are shifted (rotated) by one such that $c_1$ is paired with $B_{n-1}^2$, $c_2$ is paired with $B_{n-1}^3$, and so on. In general, for $1\le i\le m$,

$$\begin{aligned} B_n^i=\left( c_1\circ B_{n-1}^i\right) \sqcup \left( c_2\circ B_{n-1}^{i+1}\right) \sqcup \cdots \sqcup \left( c_{m-i+1} \circ B_{n-1}^m\right) \sqcup \left( c_{m-i+2} \circ B_{n-1}^1\right) \sqcup \cdots \sqcup \left( c_m\circ B_{n-1}^{i-1}\right) . \end{aligned}$$

Examples of this partition for $\Sigma =\{$A, C, G, T$\}$ and $n=2, 3$ are shown below.

$$\begin{aligned} B_2^1&=\{\text {AA, CC, GG, TT}\}\\ B_2^2&=\{\text {AC, CG, GT, TA}\}\\ B_2^3&=\{\text {AG, CT, GA, TC}\}\\ B_2^4&=\{\text {AT, CA, GC, TG}\}\\ \end{aligned}$$

$$\begin{aligned} B_3^1&=\{\text {AAA, ACC, AGG, ATT, CAC, CCG, CGT, CTA, }\text {GAG, GCT, GGA, GTC, TAT, TCA, TGC, TTG}\}\\ B_3^2&=\{\text {AAC, ACG, AGT, ATA, CAG, CCT, CGA, CTC, }\text {GAT, GCA, GGC, GTG, TAA, TCC, TGG, TTT}\}\\ B_3^3&=\{\text {AAG, ACT, AGA, ATC, CAT, CCA, CGC, CTG, }\text {GAA, GCC, GGG, GTT, TAC, TCG, TGT, TTA}\}\\ B_3^4&=\{\text {AAT, ACA, AGC, ATG, CAA, CCC, CGG, CTT, }\text {GAC, GCG, GGT, GTA, TAG, TCT, TGA, TTC}\}\\ \end{aligned}$$

Note that each sequence in $\mathcal {S}_n$ appears in exactly one of the subsets $B_n^i$, justifying the use of the disjoint union notation. (The induction proof of this claim has identical structure as the following proofs of Lemma 10 and 11, so we leave it out for conciseness.) Now we prove the correctness of this construction.

#### Lemma 10

Each constructed $B_n^i$ is a minimum (1, 1)-guaranteed subset of $\mathcal {S}_n$.

#### Proof

By Lemma 9, we only need to show that each $B_n^i$ is (1, 1)-guaranteed and has size $|\Sigma |^{n-1} = m^{n-1}$. The proof is by induction on *n*. The base case $\mathcal {S}_1=\{c_1\}\sqcup \cdots \sqcup \{c_m\}$ is easy to verify.

As the induction hypothesis, suppose that $\mathcal {S}_{n-1}=\bigsqcup _{j=1}^m B_{n-1}^j$, where each $B_{n-1}^j$ is (1, 1)-guaranteed and has size $m^{n-2}$. Consider an arbitrary index $1\le i\le m$. By construction, we have $\left| B_n^i\right| =\sum _{j=1}^m \left| B_{n-1}^j\right| = m^{n-1}$. To show that $B_n^i$ is (1, 1)-guaranteed, consider two sequences $\varvec{s}, \varvec{t}\in \mathcal {S}_n$ with $\text {edit}\left( \varvec{s}, \varvec{t}\right) =1$. If the single substitution happens on the first character, let $\varvec{x}\in \mathcal {S}_{n-1}$ be the common $(n-1)$-suffix of $\varvec{s}$ and $\varvec{t}$. Since $\bigsqcup _{j=1}^m B_{n-1}^j$ is a partition of $\mathcal {S}_{n-1}$, $\varvec{x}$ must appear in one of the subsets $B_{n-1}^{\ell }$. In $B_n^i$, it is paired with one of the characters $c_k$. Let $\varvec{y}=c_k\circ \varvec{x}$, then $\varvec{y}\in B_n^i$. Furthermore, $\varvec{s}$ and $\varvec{t}$ can each be transformed to $\varvec{y}$ by at most one substitution on the first character. Thus, $\varvec{y}\in N_n^1(\varvec{s})\cap N_n^1(\varvec{t})\cap B_n^i$.

If the single substitution between $\varvec{s}$ and $\varvec{t}$ does not happen on the first position, then they share the common first character $c_k$. In $B_n^i$, $c_k$ is paired with one of the subsets $B_{n-1}^{\ell }$. Let $\varvec{s'}$ and $\varvec{t'}$ be $(n-1)$-suffixes of $\varvec{s}$ and $\varvec{t}$, respectively. It is clear that $\text {edit}\left( \varvec{s'}, \varvec{t'}\right) =1$. By the induction hypothesis, $B_{n-1}^{\ell }$ is (1, 1)-guaranteed. So there is a sequence $\varvec{x}\in B_{n-1}^{\ell }$ of length $n-1$ such that $\text {edit}\left( \varvec{s'}, \varvec{x}\right) \le 1$ and $\text {edit}\left( \varvec{t'}, \varvec{x}\right) \le 1$. Let $\varvec{y}=c_k\circ \varvec{x}$, then $\varvec{y}\in B_n^i$ by the construction. Furthermore, $\text {edit}\left( \varvec{s}, \varvec{y}\right) =\text {edit}\left( \varvec{s'}, \varvec{x}\right) \le 1$ and $\text {edit}\left( \varvec{t}, \varvec{y}\right) =\text {edit}\left( \varvec{t'}, \varvec{x}\right) \le 1$. Thus, $\varvec{y}\in N_n^1(\varvec{s})\cap N_n^1(\varvec{t})\cap B_n^i$. Therefore, $B_n^i$ is (1, 1)-guaranteed. Since the index *i* is arbitrary, this completes the proof. $\square$

It remains to show that for each $\varvec{s}\in \mathcal {S}_n$, $\left| N_n^1(\varvec{s})\cap B_n^i\right|$ matches the lower bound in Lemma 9. Together with Lemma 10, this proves that each constructed $B_n^i$ yields an optimal (1, 3)-sensitive bucketing function in terms of minimizing both the total number of buckets and the number of buckets each length-*n* sequence is sent to.

#### Lemma 11

For $\varvec{s}\in \mathcal {S}_n$, each constructed $B_n^i$ satisfies

$$\begin{aligned} \left| N_n^1(\varvec{s})\cap B_n^i\right| = {\left\{ \begin{array}{ll} 1 &{} \text {if } \varvec{s}\in B_n^i\\ n &{} \text {if } \varvec{s}\not \in B_n^i \end{array}\right. }. \end{aligned}$$

#### Proof

We proceed by induction on *n*. The base case $n=1$ is trivially true because $|B_1^i|=1$ and all single-character sequences are within one edit of each other. Suppose that the claim is true for $n-1$. Consider an arbitrary index *i*. If $\varvec{s}\in B_n^i$, we show that any other length-*n* sequence $\varvec{t}\in B_n^i$ has edit distance at least 2 from $\varvec{s}$, namely $N_n^1(\varvec{s})\cap B_n^i=\{\varvec{s}\}$. Let $\varvec{s'}$ and $\varvec{t'}$ be the $(n-1)$-suffixes of $\varvec{s}$ and $\varvec{t}$ respectively. According to the construction, if $\varvec{s}$ and $\varvec{t}$ have the same first character, then $\varvec{s'}$ and $\varvec{t'}$ are in the same $B_{n-1}^j$ for some index *j*. By the induction hypothesis, $\text {edit}\left( \varvec{s'}, \varvec{t'}\right) \ge 2$ (otherwise $\left| N_{n-1}^1\left( \varvec{s'}\right) \cap B_{n-1}^j\right| \ge 2$), and therefore $\text {edit}\left( \varvec{s}, \varvec{t}\right) =\text {edit}\left( \varvec{s'}, \varvec{t'}\right) \ge 2$. If $\varvec{s}$ and $\varvec{t}$ are different at the first character, then $\varvec{s'}$ and $\varvec{t'}$ are not in the same $B_{n-1}^j$, so $\varvec{s'}\ne \varvec{t'}$ (recall that $B_{n-1}^j$ and $B_{n-1}^{k}$ are disjoint if $j\ne k$), namely $\text {edit}\left( \varvec{s'}, \varvec{t'}\right) \ge 1$. Together with the necessary substitution at the first character, we have $\text {edit}\left( \varvec{s}, \varvec{t}\right) =1+\text {edit}\left( \varvec{s'}, \varvec{t'}\right) \ge 2$.

If $\varvec{s}\not \in B_n^i$, Lemma 9 and 10 guarantee that $\varvec{s}$ has at least *n* 1-neighbors $\varvec{v}^{k}$ in $B_n^i$, $k=1,\ldots , n$, where $\varvec{v}^{k}$ is obtained from $\varvec{s}$ by a single substitution at position *k*. Let $\varvec{t}\ne \varvec{s}$ be a 1-neighbor of $\varvec{s}$. Since $\varvec{t}$ can only differ from $\varvec{s}$ by a single substitution at some position $\ell$, we know that either $\varvec{t}=\varvec{v}^{\ell }$ or the edit distance between $\varvec{t}$ and $\varvec{v}^{\ell }$ is 1. In the latter case, $\varvec{t}$ cannot be in $B_n^i$ otherwise $\left| N_n^1\left( \varvec{v}^{\ell }\right) \cap B_n^i\right| \ge 2$, contradicting the result of the previous paragraph. Therefore, $N_n^1(\varvec{s})\cap B_n^i=\left\{ \varvec{v}^1,\ldots \varvec{v}^{n}\right\}$ which has size *n*. $\square$

We end this section by showing that a membership query can be done in *O*(*n*) time on the (1, 1)-guaranteed subset *B* constructed above (i.e., $B=B_n^i$ for some *i*). Thanks to its regular structure, the query is performed without explicit construction of *B*. Consequently, the bucketing functions using *B* can be computed without computing and storing this subset of size $|\Sigma |^{n-1}$.

Specifically, suppose that we choose $B=B_n^i$ for some fixed $1\le i\le m$. Let $\varvec{s}$ be a given length-*n* sequence; we want to query if $\varvec{s}$ is in *B* or not. This is equivalent to determining whether the index of the partition of $\mathcal {S}_n$ that $\varvec{s}$ falls into is *i* or not. Write $\varvec{s}=s_1s_2\ldots s_n$ and let $\varvec{s}'=s_2\ldots s_n$ be the $(n-1)$-suffix of $\varvec{s}$. Suppose that it has been determined that $\varvec{s}'\in B_{n-1}^j$ for some index $1\le j\le m$, i.e., the sequence $\varvec{s}'$ of length $n-1$ comes from the *j*-th partition of $\mathcal {S}_{n-1}$. By construction, the index $\ell$ for which $\varvec{s}\in B_n^{\ell }$ is uniquely determined by the character $s_1=c_k\in \Sigma$ and the index *j* according to the formula $\ell = (j+m+1-k)\bmod m$. The base case $n=1$ is trivially given by the design that $c_p\in B_1^p$ for all $1\le p\le m$. This easily translates into a linear-time algorithm that scans the input length-*n* sequence $\varvec{s}$ backwards and compute the index $\ell$ such that $\varvec{s}\in B_n^{\ell }$. To answer the membership query, we only need to check whether $\ell =i$. We provide an implementation of both the construction and the efficient membership query of a (1, 1)-guaranteed subset at [25].

### A (3, 5)-sensitive bucketing function

Let $B\subset \mathcal {S}_n$ be one of the constructed (1, 1)-guaranteed subsets. Recall that the resulting bucketing function $f^r_B$ is $(r, 2r + 1)$-sensitive for all integers $r\ge 1$; in particular, $f^2_B$ is (2, 5)-sensitive. We are able to strengthen this result by showing that $f^2_B$ is in fact (3, 5)-sensitive.

#### Theorem 2

Let $B\subset \mathcal {S}_n$ be a (1, 1)-guaranteed subset. The bucketing function $f^2_B$ is (3, 5)-sensitive.

#### Proof

As $f_B^r$ is already proved to be (2, 5)-sensitive, to show it is (3, 5)-sensitive, we just need to prove that, for any two sequences $\varvec{s}, \varvec{t}\in \mathcal {S}_n$ with $\text {edit}\left( \varvec{s}, \varvec{t}\right) =3$, $f^2_B(\varvec{s})\cap f^2_B(\varvec{t}) = N^2_n(\varvec{s}) \cap N^2_n(\varvec{t}) \cap B \ne \emptyset$. If the three edits are all substitutions, then there are length-*n* sequences $\varvec{x}$ and $\varvec{y}$ such that $\text {edit}\left( \varvec{s}, \varvec{x}\right) =\text {edit}\left( \varvec{x}, \varvec{y}\right) =\text {edit}\left( \varvec{y}, \varvec{t}\right) =1$. Since *B* is (1, 1)-guaranteed, there is a length-*n* sequence $\varvec{z}\in B$ with $\text {edit}\left( \varvec{x}, \varvec{z}\right) \le 1$ and $\text {edit}\left( \varvec{y}, \varvec{z}\right) \le 1$. By triangle inequality, $\text {edit}\left( \varvec{s}, \varvec{z}\right) \le \text {edit}\left( \varvec{s}, \varvec{x}\right) +\text {edit}\left( \varvec{x}, \varvec{z}\right) \le 2$; $\text {edit}\left( \varvec{t}, \varvec{z}\right) \le \text {edit}\left( \varvec{t}, \varvec{y}\right) +\text {edit}\left( \varvec{y}, \varvec{z}\right) \le 2$. So $\varvec{z}\in N^2_n(\varvec{s}) \cap N^2_n(\varvec{t}) \cap B$.

If the three edits are one substitution and a pair of indels, then there is a length-*n* sequence $\varvec{x}$ such that $\text {edit}\left( \varvec{s}, \varvec{x}\right) =1$ and $\text {edit}\left( \varvec{x}, \varvec{t}\right) =2$ where the two edits between $\varvec{x}$ and $\varvec{t}$ can only be achieved by one insertion and one deletion. Let *i* be the position in $\varvec{x}$ where the deletion between $\varvec{x}$ and $\varvec{t}$ takes place. Let $\varvec{y}$ be a length-*n* sequence obtained from $\varvec{x}$ by a substitution at position *i*, so $\text {edit}\left( \varvec{x}, \varvec{y}\right) =1$. Since *B* is (1, 1)-guaranteed, there is a length-*n* sequence $\varvec{z}\in B$ with $\text {edit}\left( \varvec{x}, \varvec{z}\right) \le 1$ and $\text {edit}\left( \varvec{y}, \varvec{z}\right) \le 1$. Then $\text {edit}\left( \varvec{s}, \varvec{z}\right) \le \text {edit}\left( \varvec{s}, \varvec{x}\right) + \text {edit}\left( \varvec{x}, \varvec{z}\right) \le 2$. Observe that $\varvec{x}$ and $\varvec{z}$ differ by at most one substitution at position *i*, which will be deleted when transforming to $\varvec{t}$. So the two edits from $\varvec{x}$ to $\varvec{t}$ can also transform $\varvec{z}$ to $\varvec{t}$, namely, $\text {edit}\left( \varvec{t}, \varvec{z}\right) \le 2$. Thus, $\varvec{z}\in N^2_n(\varvec{s}) \cap N^2_n(\varvec{t}) \cap B$. $\square$

## Summary of proved LSB functions

We proposed two sets of LSB functions and studied the efficiency of them in terms of |*B*|, the total number of buckets, and $|f(\varvec{s})|$, the number of buckets a specific length-*n* sequence $\varvec{s}$ occupies. The results are summarized in Table 1.

## Experimental results on the gapped LSB functions

**Fig. 1:**
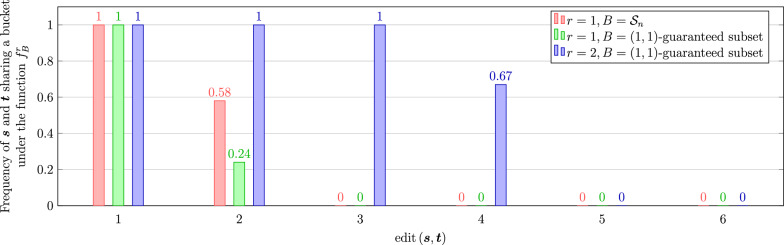
Probabilities (estimated by frequencies) that two sequences share a bucket with respect to their edit distance under three gapped LSB functions (red, green, and blue bars correspond to the rows 2–4 of Table 1)

Now we experimentally investigate the behavior of the gapped LSB functions at their respective gaps. We pick 3 LSB functions to experiment, corresponding to the rows 2–4 in Table 1. For $d=1, 2, \ldots , 6$, we generate 100, 000 random pairs $(\varvec{s}, \varvec{t})$ of sequences of length 20 with edit distance *d*. Each one of the picked LSB functions $f^r_B$ is applied and the number of pairs that share a bucket under $f^r_B$ is recorded. The code can be found at [25]. The results are shown in Fig. 1.

**Fig. 2:**
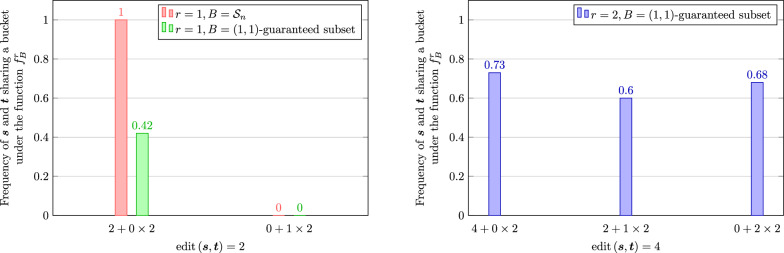
Probabilities (estimated by frequencies) that two sequences share a bucket with respect to their edit type under three gapped LSB functions. The types of edits are labeled in the format $a+b\times 2$ where *a* is the number of substitutions and *b* is the number of pairs of indels. Left: two (1, 3)-sensitive bucketing functions (rows 2 and 3 of Table 1). Right: the (3, 5)-sensitive bucketing function (row 4 of Table 1)

Recall that Lemma 6 implies $f^r_{\mathcal {S}_n}$ is $(2r-1, 2r+1)$-sensitive when *r* is odd. The discussion after the proof shows that the gap at 2*r* indeed exists. In particular, if $\varvec{s}$ can only be transformed to $\varvec{t}$ by *r* pairs of indels, then $N_n^r(\varvec{s})\cap N_n^r(\varvec{t})=\emptyset$. On the other hand, if there are some substitutions among the 2*r* edits between $\varvec{s}$ and $\varvec{t}$, then by a similar construction as in the case where *r* is even, we can find a length-*n* sequence $\varvec{v}$ such that $\text {edit}\left( \varvec{s}, \varvec{v}\right) =\text {edit}\left( \varvec{v}, \varvec{t}\right) =r$. Motivated by this observation, we further explore the performance of the LSB functions at the gap for different types of edits. Given a gapped LSB function *f*, for the gap at *d*, define categories $0,\ldots ,\lfloor d/2\rfloor$ corresponding to the types of edits: a pair of length-*n* sequences with edit distance *d* is in the *i*-th category if they can be transformed to each other with *i* pairs of indels (and $d-2i$ substitutions) but not $i-1$ pairs of indels (and $d-2i+2$ substitutions). Figure 2 shows the results for the three LSB functions in Fig. 1 at their respective gaps with respect to different types of edits. Observe that the result for $f^1_{\mathcal {S}_n}$ (in red) agrees with our analysis above.

## Conclusions

We introduce locality-sensitive bucketing (LSB) functions, that generalize locality-sensitive hashing (LSH) functions by allowing it to map a sequence into multiple buckets. This generalization makes the LSB functions easier to construct, while guaranteeing high sensitivity and specificity in a deterministic manner. We construct such functions, prove their properties, and show that some of them are optimal under proposed criteria. We also reveal several properties and structures of the metric space $\mathcal {S}_n$, which are of independent interests for studying LSH functions and the edit distance.

Our results for LSB functions can be improved in several aspects. An obvious open problem is to design $(d_1, d_2)$-sensitive functions that are not covered here. For this purpose, one direction is to construct optimal (*r*, *r*)-guaranteed subsets for $r>1$. As an implication of Lemma 11, it is worth noting that the optimal (1, 1)-guaranteed subset is a maximal independent set in the undirected graph $G_n^1$ whose vertex set is $\mathcal {S}_n$ and each sequence is connected to all its 1-neighbors. It is natural to suspect that similar results hold for (*r*, *r*)-guaranteed subsets with larger *r*. Another approach is to use other more well-studied sets as buckets and define LSB functions based on their connections with $\mathcal {S}_n$. This is closely related to the problem of embedding $\mathcal {S}_n$ which is difficult as noted in the introduction. Our results suggest a new angle to this challenging problem: instead of restricting our attention to embedding $\mathcal {S}_n$ into metric spaces, it may be beneficial to consider a broader category of spaces that are equipped with a non-transitive relation (here in LSB functions we used subsets of integers with the “have a nonempty intersection” relation). Yet another interesting future research direction would be to explore the possibility of improving the practical time and space efficiency of computing and applying LSB functions.

A technique commonly used to boost the sensitivity of an LSH function is known as the OR-amplification. It combines multiple LSH functions in parallel, which can be viewed as sending each sequence into multiple buckets such that the probability of having similar sequences in one bucket is higher than using the individual functions separately. However, as a side effect, the OR-amplification hurts specificity: the chance that dissimilar sequences share a bucket also increases. It is therefore necessary to combine it with other techniques and choosing parameters to balance sensitivity and specificity is a delicate work. On contrast, the LSB function introduced in this paper achieves a provably optimal separation of similar and dissimilar sequences. In addition, the OR-amplification approach can also be applied on top of the LSB functions as needed.

## Data Availability

Not applicable.

## Abbreviations

LSH: Locality-sensitive hashing
LSB: Locality-sensitive bucketing
